# Cox regression can be collapsible and Aalen regression can be non-collapsible

**DOI:** 10.1007/s10985-022-09578-0

**Published:** 2022-10-21

**Authors:** Sven Ove Samuelsen

**Affiliations:** Department of Mathematics, University of Oslo, P.O.1053, 0316 Blindern, Oslo, Norway

**Keywords:** Additive hazards models, Instrumental variables, Linear hazards models, Matched cohort study, Proportional hazards models, Randomized clinical study

## Abstract

It is well-known that the additive hazards model is collapsible, in the sense that when omitting one covariate from a model with two independent covariates, the marginal model is still an additive hazards model with the same regression coefficient or function for the remaining covariate. In contrast, for the proportional hazards model under the same covariate assumption, the marginal model is no longer a proportional hazards model and is not collapsible. These results, however, relate to the model specification and not to the regression parameter estimators. We point out that if covariates in risk sets at all event times are independent then both Cox and Aalen regression estimators are collapsible, in the sense that the parameter estimators in the full and marginal models are consistent for the same value. Vice-versa, if this assumption fails, then the estimates will change systematically both for Cox and Aalen regression. In particular, if the data are generated by an Aalen model with censoring independent of covariates both Cox and Aalen regression is collapsible, but if generated by a proportional hazards model neither estimators are. We will also discuss settings where survival times are generated by proportional hazards models with censoring patterns providing uncorrelated covariates and hence collapsible Cox and Aalen regression estimates. Furthermore, possible consequences for instrumental variable analyses are discussed.

**Supplementary Information:**

The online version contains supplementary material available at 10.1007/s10985-022-09578-0.

## Introduction

The concept of collapsibility was discussed by Whittemore (1978) who investigated conditions for when inference from lower dimensional contingency tables would be the same as from high dimensional. Later on it has been used in the context of regression models as the situation where removal of some covariates, independent of the remaining, will not change the regression parameters of the remaining covariates in the marginal model. Collapsibility is of relevance for causal modeling (Greenland et al. 1999). A context where collapsibility is important is a trial where exposure is randomized and thereby made independent of otherwise potentially confounding variables which may be unobserved. Collapsibility is then the condition for unbiased estimation of a common exposure effect from such a randomized study when the other variables are not taken into account and there is no interaction between them and the exposure (Gail et al. 1984). Furthermore, for handling unobservable confounding by means of instrumental variables (Tchetgen Tchetgen et al. 2015), the concept of collapsibility is important.

Gail et al. (1984) demonstrated that for generalized linear models the collapsibility property holds with identity and log-links and that these essentially are the only links giving collapsibility and so for instance logistic regression is non-collapsible. For survival analysis it was demonstrated by several authors that with the proportional hazards model, removal of an independent covariate gives attenuation towards zero for regression parameters of the remaining covariates (Struthers and Kalbfleisch 1986; Solomon 1984; Bretagnolle and Huber-Carol 1988; Gail et al. 1984). In contrast, for the additive hazards model (Aalen 1980) an appendix of Aalen (1989) demonstrates the collapsibility property.

In this paper we will distinguish between collapsible models and collapsible estimators. By a collapsible model we will mean that the marginal model after integrating out some covariates that are independent of the remaining will have the same regression coefficients for the remaining covariates. A collapsible estimator is similarly an estimator which is consistent for the same value in the full and the marginal models, and so the estimate does not systematically change after removing independent covariates from the model.

Sometimes both models and estimators are collapsible, for instance (Gail et al. 1984) showed that maximum likelihood and moment estimators are collapsible under generalized linear models with identity and log-links. However, an estimator need not be collapsible even if the model is collapsible and it can be possible that an estimator is collapsible even if the model is not. A simple example is least squares estimators which are collapsible when covariates are uncorrelated irrespectively of whether the data generating model is collapsible.

We will specifically in this paper discuss survival data and show that even if the additive hazards model is collapsible, estimators under this model can be non-collapsible under situations that will be discussed. By a flip of the coin Cox-estimators can be collapsible even if the proportional hazards model is not.

As a background for the results we refer to Aalen et al. (2015) who pointed out that under an additive hazards model covariates that are independent at the outset will continue to be so at all later times among individuals that have not yet experienced the event of interest. They also showed that this in fact is equivalent with an additive hazards model specification. Thus under other hazard specifications such as the proportional hazards model a dependence between the covariates will develop as time evolves and non-collapsibility of these models can be explained from this perspective. However, if postulating that covariates are independent in all risk sets then also Cox regression will become collapsible, as will be demonstrated.

Independence in all risk sets can arise in several ways. Our first example is that the data was generated by an additive hazards model, but very possibly the researchers were unaware of this and perhaps by convention chose Cox regression for the data analysis. But it is also possible that right censoring or left truncation patterns had a structure maintaining independence over risk sets. Other settings that may lead to independence of covariates over risk sets are commented on in Sect. 2 and in the Discussion section.

Such schemes can be considered artificial, but still knowledge of the fact is useful. For instance it has been noted that proportional hazards models are approximately collapsible with low incidence which can be understood by that the model induced dependence can then not have developed extensively.

Also, it is very likely that the data are not exactly generated by an additive hazards model and so dependence between covariates may develop which can give rise to non-collapsible estimation in such models. Furthermore censoring, for instance by a competing risk, may generate dependence between covariates leading to non-collapsible estimation. Dependence and non-collapsible estimation in additive hazards models could also arise from for instance left-truncation.

In the next section we set up the framework for collapsible and non-collapsible models and estimators and specify this within a survival analysis framework. We then demonstrate that additive hazards models are collapsible, whereas proportional hazards models are non-collapsible. Furthermore, the condition for the (standard) estimators under these models to be collapsible, namely that covariates are independent in all risk sets, is derived. In Sect. 3 these properties are studied by means of data simulated from additive and proportional hazards models with censoring independent and dependent on covariates and analyzed by Cox regression and regression methods for additive hazards. Following in the section we consider instrumental variables estimation under both the additive and proportional hazards models and give examples demonstrating that under both models valid estimation requires that the instrument and the unknown confounders are independent in all risk sets. The paper is rounded off with a short discussion section.

## Models and main results

### Collapsibility in general

Assume that a response *Y* depends on covariates $Z=(Z_1,Z_2)$ where in general $Z_j$ can be vectors of length $p_j, j=1,2$. However, for presentational ease we let $p_1=p_2=1$ and so the $Z_j$ are scalars. The general results with one or both $p_j > 1$ are obvious extensions. We consider the $Z_j$ as random and assume that $Z_1$ and $Z_2$ are independent. Conditional on $Z=z=(z_1,z_2)$ the distribution of *Y* is given as $f(y | z) = f_0(y; \theta , \beta _1 z_1+\beta _2 z_2)$ for some distribution function $f_0(y; \theta , \eta )$. The model is then collapsible if the distribution of *Y* only given $Z_1=z_1$ can be written as $f_1(y; \gamma , \beta _1 z_1)$ with the same $\beta _1$ as in $f_0(y; \theta , \beta _1 z_1+\beta _2 z_2)$ and non-collapsible if not.

An estimator $\hat{\beta }_1$ of $\beta _1$ based on a model specification of *Y* conditional only on the covariate $Z_1=z_1$ is collapsible if $\hat{\beta }_1$ is consistent for the same $\beta _1$ as in the specification $f_0(y; \theta , \beta _1 z_1+\beta _2 z_2)$. With a consistent estimator $(\tilde{\beta }_1,\tilde{\beta }_2)$ under this specification we thus have that there is no a systematic difference between $\hat{\beta }_1$ and $\tilde{\beta }_1$.

### Survival framework

We will be concerned with survival models and survival data. The models are specified by the hazard functions and in particular the proportional hazards model is given as the hazard with covariate *z* (Cox 1972; Aalen et al. 2008),

$$\begin{aligned} \lambda (t;z) = \lambda _0(t) \exp (\beta 'z) = \lambda _0(t) \exp ( \beta _1 z_1+\beta _2 z_2) \end{aligned}$$

for some baseline hazard function $\lambda _0(t)$ with $z_j=0, j=1,2$, $\beta =(\beta _1,\beta _2)'$ and a regression vector *z*. In contrast the additive hazards model of Aalen (1989) is written as

$$\begin{aligned} \lambda (t;z) = \beta _0(t) + \beta (t)'z = \beta _0(t) + \beta _1(t) z_1+ \beta _2(t) z_2 \end{aligned}$$

where $\beta _0(t)$ is also a baseline hazard function and $\beta _j(t), j=1,2$ regressions functions corresponding to the *j*-th component of *z*. Other specifications of the additive hazards model are given by setting $\beta _j(t)=\beta _j$ as a fixed parameter for both *j* (Lin and Ying 1994) and only for one components of $\beta (t)$ (McKeague and Sasieni 1994). We will in several simulations consider the regression parameter formulation of Lin and Ying because it corresponds to the Cox model in parametrization with a baseline hazard function $\lambda _0(t)$ or $\beta _0(t)$ and a linear predictor $\beta _1 z_1+\beta _2 z_2$. A thorough treatment of additive hazards model can be found in Martinussen and Scheike (2006) and these models can be fitted using their R-library timereg.

### Collapsibilty of additive hazards models

The survival function corresponding to the additive hazards model considering the covariates $Z=(Z_1,Z_2)$ as random becomes

$$\begin{aligned} S(t| Z) = \exp \left( - \int _0^t \lambda (s;Z) ds\right) = \exp \left( -B_0(t) - B_1(t) Z_{1}- B_2(t) Z_{2}\right) \end{aligned}$$

with cumulative regression functions $B_j(t)=\int _0^t \beta _j(s) ds, j=0,1,2$. Thus conditioning on only $Z_1$ we obtain

$$\begin{aligned} \begin{array}{ll} S(t| Z_1) = \text{ E } [S(t| Z) | Z_1 ] &{} = \int \exp (-B_0(t) - B_1(t) Z_{1}- B_2(t) z_{2}) g_2(z_2 | Z_1) dz_2 \\ \\ &{} = \exp (-B_0(t) - B_1(t) Z_{1}) \int \exp (- B_2(t) z_{2}) g_2(z_2 ) dz_2 \end{array} \end{aligned}$$

where the conditional density of $Z_2 | Z_1$ is $g_2(z_2 | Z_1) = g_2(z_2)$ due to the independence between the $Z_j$. The hazard corresponding to this conditional survival function equals $\gamma _0(t) + \beta _1(t) z_1$ where the baseline hazard is given by $\gamma _0(t)=\beta _0(t)-\frac{d}{dt}\log (\int \exp (- B_2(t) z_{2}) g_2(z_2 ) dz_2)$. This is an additive hazards model with regression function $\beta _1(t)$ and baseline hazard $\gamma _0(t)$, thus the additive hazards model is collapsible. Note that the result holds also when one or both of the $\beta _j(t)=\beta _j$ are constant.

Collapsibility also holds for a more general version of the additive model where terms $\beta _j(t) z_j$ are extended to regression functions $\beta _j(t;z_j)$ and cumulative regression functions $B_j(t,z_j) = \int _0^t \beta _j(s;z_j) ds$. For this model we get that the density $g(z_1,z_2;t)$ of $(Z_1,Z_2)$ conditional on the survival time $T>t$ can be written as

$$\begin{aligned} g(z_1,z_2;t) \propto g(z_1,z_2) \exp (-B_0(t) - B_1(t, z_{1})- B_2(t, z_{2})) \end{aligned}$$

and so we can write $g(z_1,z_2;t)=g_1(z_1;t)g_2(z_1;t)$ for marginal conditional densities $g_j(z_j;t)$ of $Z_j$ given $T>t$. Thus under additive hazards models $Z_1$ and $Z_2$ are independent given $T>t$ for all *t*. As pointed out by Aalen et al. (2015) this property will only hold under the generalized additive hazards model with regression functions $\beta _j(t;z_j)$.

### Non-collapsibilty of proportional hazards models

In contrast for the proportional hazards assumption we get the survival function of $T>t$ given $Z=(Z_1,Z_2)$ as

$$\begin{aligned} S(t| Z) = \exp \left( - \int _0^t \lambda (s;Z) ds\right) = \exp \left( -\Lambda _0(t) \exp (\beta _1 Z_1+\beta _2 Z_2)\right) . \end{aligned}$$

where $\Lambda _0(t) = \int _0^t \lambda _0(s) ds$ and the marginal survival function only given $Z_1$ becomes

$$\begin{aligned} S(t | Z_1) = \text{ E } [S(t| Z) | Z_1 ] = \int \exp \left( -\Lambda _0(t) \exp (\beta _1 Z_1+\beta _2 z_2)\right) g_2(z_2 ) dz_2 \\ \end{aligned}$$

which can not be written as a survival function under a proportional hazards model and so, as several authors has demonstrated (Struthers and Kalbfleisch 1986; Solomon 1984; Bretagnolle and Huber-Carol 1988), the proportional hazards model is not collapsible. Also note that since the proportional hazards model is different from the additive hazards model, a dependence between $Z_1$ and $Z_2$ among individuals with survival $T>t$ will develop as time *t* increases when data were generated under a proportional hazards model with the $Z_j$ independent at time $t=0$.

### Collapsibilty of estimators from additive hazards models

Regarding estimation, the additive hazards models are usually fitted with least squares techniques. The most commonly used method, suggested by Aalen (1980), Aalen (1989), consists in estimating the cumulative regression functions $B_j(t) = \int _0^t \beta _j(s) ds$ as a sum of increments at each event time $t_j$ where the increments are the least squares solution using indicators of event $dN_i(t_j)$ of individual *i* as responses with a design matrix consisting of the covariates of individuals at risk at that time. If the $Z_j$ were independent at the outset and the data were generated by the additive hazards model, the $Z_j$ among those who have not yet experienced the event will be independent. Also, if censoring is independent of covariates the $Z_j$ will continue to be independent. A basic fact about least squares estimators is that one can remove uncorrelated covariates without changing the estimate for the remaining covariates. Thus in this situation the increment estimates do not change systematically and so the estimator of the cumulative regression function is collapsible. This property will also hold, as commented in more detail on in the end of this subsection, if censoring depend on covariates according to an additive hazards model since then the overall model for leaving the risk sets will follow an additive hazards model and the covariates will continue to be independent and uncorrelated.

With the specification of the additive model with some or all $\beta _j(t)=\beta _j$ constant the estimators suggested by Lin and Ying (1994) and by McKeague and Sasieni (1994) were presented as a two step procedure where the constant $\beta _j$ are estimated first and the non-constant $\beta _0(t)$ and $\beta _{j}(t)$ in the next step.

Specifically the Lin-Ying estimator of $\beta =(\beta _1,\beta _2)'$ is given as

$$\begin{aligned} \hat{\beta }= [\sum _{i=1}^n \int Y_i(t) (Z_i- \bar{Z}(t))^{\otimes 2} dt ]^{-1} \sum _{i=1}^n \int (Z_i- \bar{Z}(t)) dN_i(t) \end{aligned}$$

where $Z_i=(Z_{i1},Z_{i2})'$, $Y_i(t)$ the indicator that individual *i* is at risk at $t-$, $dN_i(t)$ and indicator of event of individual *i* at time *t*, $\bar{Z}(t) = \sum _{i=1}^n Z_i Y_i(t) / Y(t)$ with $Y(t)=\sum _{i=1}^n Y_i(t)$ and $a^{\otimes 2} = a a'$. It then follows that one can not remove one covariate from the model and retain exactly the same estimate for the remaining covariate when

$$\begin{aligned} \sum _{i=1}^n \int Y_i(t) (Z_{i1}- \bar{Z}_1(t)) (Z_{i2}- \bar{Z}_2(t)) dt \ne 0 \end{aligned}$$

In a finite sample there will practically always be a slight change in the estimate of $\beta _1$ when excluding $z_{i2}$ from the model. However, when $Z_1$ and $Z_2$ are independent the correlations between $z_{i1}$ and $z_{i2}$ among those at risk at different event times $t_k$ will tend to zero and the estimates of $\beta _1$ in the full and marginal model will be consistent for the same value.

As previously mentioned, independence between the covariates will cease to be if the data generating mechanism is not an additive hazards model or if censoring or truncation forces the distribution of the observed $z_1$ and $z_2$ in different risk set to be correlated. In particular for the right-censoring situation we have that the times to event and censoring are typically assumed independent given covariates $z_1$ and $z_2$ with hazards $\lambda (t,z)=\beta _0(t) + \beta _1(t,z_1)+\beta _2(t,z_2)$ for the event time and $\lambda _C(t,z)$ for the censoring time. Then the censored survival time, i.e. the minimum of the event time and the censoring time, has a hazard $\lambda (t,z)+\lambda _C(t,z)$ which is an additive hazard model if and only if the hazard for censoring $\lambda _C(t,z)=\beta _{0C}(t) + \beta _{1C}(t,z_1)+\beta _{2C}(t,z_2)$, i.e. is an additive hazards model. From Aalen et al. (2015) it then follows that the covariates will be independent at all event times. We can also note that this extends to a competing risk situation with all cause-specific hazards and also the censoring hazards are following additive models.

### Collapsibilty of estimators from proportional hazards models

We will now demonstrate that also Cox regression is collapsible when the covariates are independent in all risk sets. The argument is presented assuming $Z_2$ is omitted in the marginal model. The score-function of the (erroneously specified) partial likelihood with only $Z_1$ as covariate is then given as

$$\begin{aligned} U_{1}^{M}(\beta _1) = \sum _{i=1}^n \int \left[ Z_{i1}-S_M^{(1)}(\beta _1,t)/S_M^{(0)}(\beta _1,t)\right] dN_i(t) = 0 \end{aligned}$$

where $N_i(t)$ is (still) the counting process for the number of events up to time *t*, $\mathcal{R}(t)$ the risk set at this time and $S_M^{(k)}(\beta _1,t) = \sum _{i \in \mathcal{R}(t)} Z_{i1}^k \exp (\beta _1 Z_{i1}), k=0,1$. (Alternatively and perhaps typographically more pleasing we can write $S_M^{(k)}(\beta _1,t)=\sum _{i =1}^n Z_{i1}^k Y_i(t) \exp (\beta _1 Z_{i1})$ and similarly for $S_{F1}^{(1)}(\beta ,t)$ and $S_{F}^{(0)}(\beta ,t)$ below, but in this context it is useful to emphasize independence conditional on risk sets $\mathcal{R}(t))$.

Similarly the first component of the score function from the full partial likelihood (for the full model) with both covariates can be written as

$$\begin{aligned} U_{1}^F(\beta ) = \sum _{i=1}^n \int \left[ Z_{i1}-S_{F1}^{(1)}(\beta ,t)/S_{F}^{(0)}(\beta ,t)\right] dN_i(t) = 0 \end{aligned}$$

with the definitions $S_{F1}^{(1)}(\beta ,t) = \sum _{i \in \mathcal{R}(t)} Z_{i1} \exp (\beta _1 Z_{i1}+\beta _2 Z_{i2})$ and $S_{F}^{(0)}(\beta ,t) = \sum _{i \in \mathcal{R}(t)} \exp (\beta _1 Z_{i1}+\beta _2 Z_{i2})$.

When the risk set sizes grows to infinity we get for the fraction between the terms $S_M^{(1)}(\beta _1,t)$ and $S_M^{(0)}(\beta _1,t)$ of the marginal score that

$$\begin{aligned} \frac{S_M^{(1)}(\beta _1,t)}{S_M^{(0)}(\beta _1,t)} \rightarrow \frac{ \text{ E } [Z_{i1} \exp (\beta _1 Z_{i1} ) | i \in \mathcal{R}(t) ] }{ \text{ E } [ \exp (\beta _1 Z_{i1}) | i \in \mathcal{R}(t)] } = e(\beta _1,t) \end{aligned}$$

for a function $$e(\beta _1,t)$$.

In comparison for the term $S_{1F}^{(1)}(\beta ,t)/S_{F}^{(0)}(\beta ,t)$ in the first component of the full score we get

$$\begin{aligned} \frac{S_{1F}^{(1)}(\beta ,t)}{S_{F}^{(0)}(\beta ,t)} &\rightarrow \frac{ \text{ E } [Z_{i1} \exp (\beta _1 Z_{i1}+\beta _2 Z_{i2} ) | i \in \mathcal{R}(t) ] }{ \text{ E } [ \exp (\beta _1 Z_{i1} +\beta _2 Z_{i2} ) | i \in \mathcal{R}(t)] }\\ & = \frac{ \text{ E } [Z_{i1} \exp (\beta _1 Z_{i1} ) | i \in \mathcal{R}(t) ] }{ \text{ E } [ \exp (\beta _1 Z_{i1}) | i \in \mathcal{R}(t)] } = e(\beta _1,t) \end{aligned}$$

for the same function $e(\beta _1,t)$. This since $Z_{i1}$ and $Z_{i2}$ are independent in all risk sets. Thus the terms have the same limit and depend only on $\beta _1$. It then follows that the estimator of $\beta _1$ obtained from the marginal partial likelihood and the full partial likelihood will be consistent for the same value and so Cox regression is collapsible.

Collapsible Cox regression is then obtained when covariates are independent at all event times. This will be achieved under additive hazards model with initially independent covariates and censoring that is either independent of covariates or more generally following an additive hazards model. But it can also be obtained if the event time models differ from an additive hazards model, say are generated by a proportional hazards model, but the censoring mechanisms counters the dependency generated by the model and so ensuring independence between covariates at all event times.

In the simulations of Sect. 3.2.1 a censoring mechanism is developed that gives approximately uncorrelated covariates over all risk sets resulting in approximate collapsible estimation. But there actually exists one mechanism that will give exact independence. Assume that the event times follow a proportional hazards model $\lambda (t;z) = \lambda _0(t) \exp (\beta 'z) = \lambda _0(t) \exp ( \beta _1 z_1+\beta _2 z_2)$ and that the censoring times are drawn from a hazard $\lambda _C(t;z) = \beta _0(t) + \beta _1(t,z_1)+\beta _2(t,z_2) - \lambda (t;z) \ge 0$ for all possible $z_j, j=1,2$. Then the hazard of the censored survival time is given as $\lambda (t;z)+\lambda _C(t;z)$ which is an additive hazards model, and so with right censored data the covariates will be independent for all risk sets. Furthermore, with competing risk data and only right censoring, independence will be achieved if the sum of the cause-specific hazards and the hazard for the censoring is an additive hazard.

Furthermore, with left-truncation where indivudals at their event or censoring time are replaced by individuals with exactly the same covariates would lead to collapsible Cox regression. This is then related to a renewal or (Andersen and Gill 1982) process where indidividuals may return to the risk set after events. It is then also closely connected to Poisson-processes dependent on covariates through log-linear intensities for which collapsibilty follows from the results of Gail et al. (1984). Some other possible mechanism leading to independence between covariates in all risk sets are commented on in the Discussion section.

## Simulation studies

In this section we will use simulations to illustrate non-collapsible estimation under additive hazards models specifications and collapsible estimation under proportional hazards specifications.

We will first consider data generated by proportional hazards models with censoring independent of covariates and study the non-collapsibility both with Cox regression and with Aalen regression and Lin-Ying regression. These results are then contrasted with data generated by a Lin-Ying model also with censoring independent of covariates and demonstrate that there is then no systematic change using either estimators for assuming additive or proportional hazards model specification when omitting one covariate.

In a next set of simulations we will also consider survival times generated with additive or proportional hazards models, but with censoring mechanism that induces dependency between the covariates under the additive hazards specification or reduces the induced dependency under the proportional hazards specification. The effects this will have on collapsibility or non-collapsibility of Cox regression and Ling-Ying/Aalen regression is then demonstrated.

Finally instrumental variable analysis developed for additive hazards models will be considered discussing the issue of independence between the instrument and the unknown confounders in all risk sets.

### Independent censoring

#### Proportional hazards model data, independent censoring

The model for the simulation is given by the hazard for event $\lambda (t | z_1,z_2) = \lambda _0(t) \exp ( \beta _1 z_1+\beta _2 z_2)$ where $\beta _1=0.5, \beta _2=1$ and the baseline hazard $\lambda _0(t)=1$. The covariates $z_1$ and $z_2$ are uniform on [0, 2] and independent. The censoring times have a constant hazard equal to 2.2 and is independent of the covariates. This model was simulated with $n=5000$ individuals for 1000 runs. This gave a proportion of uncensored event times of 67%.

The data were then fitted with Cox regression and Lin-Ying regression both in bivariate models with $z_1$ and $z_2$ included and in univariate models with only $z_1$ included. Results for the average estimated regression parameters $\hat{\beta }_1$ are presented in Table 1.

**Table 1:** Average of $\hat{\beta }_1$ for bivariate and univariate Cox- and Lin-Ying-regressions under simulated Cox-models

Model specification	Average estimate of $\beta _1$	Univariate/bivariate
Bivariate Cox	0.498	
Univariate Cox	0.421	0.850
Bivariate Lin-Ying	2.130	
Univariate Lin-Ying	1.811	0.846

We note that with Cox regression $\beta _1$ is estimated without bias in the bivariate Cox regression with both $z_1$ and $z_2$ included. In the univariate models only including $z_1$ the average estimate drops from 0.498 to 0.421, in accordance with Cox regression being non-collapsible. This gives a fraction between the average $\hat{\beta }_1$ estimates in these two models of 0.850. For Lin-Ying regression the estimated $\hat{\beta }_1$ naturally differs, but again we see that the univariate estimates are considerably smaller than the bivariate $\hat{\beta }_1$ and the fraction between these averages are 0.846, so the degree of bias introduced by omitting $z_2$ is the same as for the Cox regression. Thus the Lin-Ying regression was also non-collapsible.

Univariate $\lambda _0(t)+\beta _1(t) z_1$ and bivariate $\lambda _0(t)+\beta _1(t) z_1+\beta _2(t) z_2$ Aalen-models were also fitted. The estimated $B_1(t)=\int _0^t \beta _1(s) ds$ for one run both bivariate with both covariates and univariate with only $z_1$ included is displayed in Panel A in Figure S1 in the supplement. The cumulative regression function is visibly smaller for the univariate model. This impression is confirmed in Panel B showing the averages of 1000 such $\hat{B}_1(t)$. The curvature of these functions is due to the simulated Cox-model and is similar to findings in Aalen (1989) and Henderson and Milner (1991).

Panel C of Figure S1 in the supplement shows how empirical correlations of $z_1$ and $z_2$ of those at risk changes as time increases both in one run and averaged over the runs. Uncertainty limits for one run $\pm 1.96/\sqrt{Y(t)}$ where *Y*(*t*) is the number at risk at time *t* are included. From initially being uncorrelated we see that a negative correlation develops, due to the Cox-model, as time increases. The average of such correlations over all runs confirms that this negative correlation is not random.

#### Additive hazards model data, independent censoring

The hazard for the data generating model is now given by the Lin-Ying model $\lambda (t | z_1,z_2) = \beta _0(t) + \beta _1 z_1+\beta _2 z_2$ where again $\beta _1=0.5$ and $\beta _2=1$, $z_1$ and $z_2$ are independent and uniformly distributed over [0, 1] and the baseline regression function equals $\beta _0(t)=1$. This is thus a Lin-Ying model with cumulative intercept function $B_0(t) = t$ and cumulative regression function $B_1(t)=0.5 t$ and $B_2(t) = t$. The censoring distribution is given by a constant hazard equal to 1. We simulated populations of size $n=5000$ and repeated this for 1000 runs. The average proportion of uncensored observations now became 65%.

The data were also as in Sect. 3.1.1 fitted with Lin-Ying regression and Cox regression under both bivariate and univariate model specifications. The average of the regression parameter estimates $\beta _1$ are given in Table 2. We note that the average parameter estimates are equal to 3 (actually 4) decimals between the bivariate and univariate models, although they naturally differ when fitting the (wrong) Cox-model or the (correct) Lin-Ying model. The simulation thus demonstrates the results that neither Lin-Ying nor Cox-regression estimates are changed systematically when excluding an independent covariates with data generated by an additive hazards model with censoring independent of covariates and hence covariates are independent in all risk sets.

**Table 2:** Average of $\hat{\beta }_1$ for bivariate and univariate Cox- and Lin-Ying-regressions under simulated Lin-Ying models

Model specification	Average estimate of $\beta _1$	Univariate/bivariate
Bivariate Cox	0.288	
Univariate Cox	0.288	1.000
Bivariate Lin-Ying	0.494	
Univariate Lin-Ying	0.494	1.000

Again Aalen models were fitted and Panel A of Figure S2 in the supplement gives estimated $B_1(t)$ in univariate and bivariate model specifications for one run, similarly Panel B gives corresponding averages over the 1000 runs. In accordance with theory there is no systematic difference between the curves in Panel A. This confirmed in Panel B where averages of the bivariate and univariate estimates are indistinguishable from each other and the true $B_1(t) = 0.5 t$.

Furthermore, Panel C gives correlations between $z_1$ and $z_2$ among individuals at risk for different *t*. The line for one run falls generally within the uncertainty limits $\pm 1.96/\sqrt{Y(t)}$ and the average over 1000 runs is very close to zero. As theory predicts no correlation will develop as time increases.

### Dependent censoring

#### Proportional hazards model data, dependent censoring

It was shown in Sect. 3 that Cox regression is collapsible if covariates are independent over all risk sets. However, when data are generated by a proportional hazards model a dependence will develop as time increases. This dependency could be counteracted on by also letting the hazard of censoring depend on a proportional hazards model.

In this section it will be demonstrated that with data generated by a proportional hazards model, but with a suitably chosen proportional hazards model also for the censoring it is possible to obtain a situation where the covariates will be approximately uncorrelated in all risk sets. It is then demonstrated that Cox regression under this correct model is approximately collapsible and the same holds true for the (wrong) Lin-Ying and Aalen models.

We use the same Cox-model as in Sect. 3.1.1 for generating the data, thus $z_j, j=1,2$, are uniform on $[0,2], \beta _1=0.5, \beta _2=1$ and $\lambda _0(t)=1$. A censoring model that for this event time model gave close to uncorrelated covariates in all risk sets, found after some tuning of parameters, was given by hazards $\lambda _C(t) = \exp (1.15 \beta _1 z_1-2 \beta _2 z_2)$ for censoring times $C_i$. This model was fitted both with bivariate and univariate Cox- and Lin-Ying models with $n=5000$ observation in 1000 simulations. The proportion uncensored observations was 67%. Results are given in Table 3 and show that both Cox regression and Lin-Ying was practically collapsible with this setting.

**Table 3:** Average of $\hat{\beta }_1$ for bivariate and univariate Cox- and Lin-Ying-regressions under simulated Cox models with dependent censoring

Model specification	Average estimate of $\beta _1$	Univariate/bivariate
Bivariate Cox	0.498	
Univariate Cox	0.495	1.007
Bivariate Lin-Ying	2.279	
Univariate Lin-Ying	2.296	0.993

As in previous sections results for fitting the Aalen model are compared between the bivariate models with both covariates and univariate models using only $z_1$. The cumulative regression functions $\hat{B}_1(t)$ in one run are shown in Figure S3 in the supplement, panel A, and the corresponding averages over 1000 simulations in Panel B. Both panels indicate approximate collapsibility under the given setting.

Panel C in Figure S3 then shows the correlations between $z_1$ and $z_2$ in different risk sets for one run with uncertainty limits and averaged over 1000 runs. This panel demonstrates that the covariates were close to uncorrelated over the risk sets.

#### Additive hazards model data, dependent censoring

We will in this section demonstrate that even when event times data are generated by an additive hazards, Lin-Ying and Aalen regressions may fail to be collapsible if the censoring times are dependent on covariates with a non-additive hazards model. For convenience we choose a proportional hazards model for the censoring times. The event times follow the same model as in Sect. 3.1.2, so the covariates $z_1$ and $z_2$ are uniform [0, 1], the additive hazards model is $\lambda (t | z_1,z_2) = \beta _0(t) + \beta _1 z_1+\beta _2 z_2$ where $\beta _1=0.5$ and $\beta _2=1$. The censoring times, though, are generated by the proportional hazards $\lambda _C(t) = \exp (-2.2+3 \beta _2 z_1+2 \beta _1 z_1)$ found after some tuning of parameters. This model was simulated with sample size $n=5000$ for 1000 simulations. The proportion of uncensored event times then became 67%.

As above both univariate and bivariate Cox-models and Lin-Ying models were fitted. Results are given in Table 4. Both Cox regression and Lin-Ying display non-collapsibility as estimates are clearly biased when $z_2$ is omitted from the model. The ratios between bivariate and univariate regression coefficients for $z_1$ and in relative terms the bias is almost equal.

**Table 4:** Average of $\hat{\beta }_1$ for bivariate and univariate Cox- and Lin-Ying-regressions under simulated Lin-Ying models with dependent censoring

Model specification	Average estimate of $\beta _1$	Univariate/bivariate
Bivariate Cox	0.296	
Univariate Cox	0.258	0.872
Bivariate Lin-Ying	0.493	
Univariate Lin-Ying	0.432	0.876

Also, as above, Aalen models where fitted with the data. Results are displayed in Figure S4 in the supplement with panel A for one run and Panel B for the average over all 1000 runs. Both panels display that the cumulative regression function $\hat{B}_1(t)$ in the model with $z_1$ only included are lower than the corresponding estimates in the model with both $z_1$ and $z_2$, this in accordance with the results in Table 4.

Furthermore, in Panel C of Figure S4 we see the correlations between the covariates over different risk sets. One clearly see that a negative correlation develops as time increases, and the bias of the regression coefficients and in $\hat{B}_1(t)$ univariate models can be explained by this dependence.

### Instrumental variables

Instrumental variables analysis is a technique allowing for adjustment for unknown confounding. The basic setup is that there is one exposure variable *X*, an unobserved confounder *U* dependent with *X*, an instrument I that is correlated with *X* but independent of *U* and an outcome variable *Y* dependent on *X* and *U*. Since *X* and *U* are dependent the estimated regression parameter of *X* alone on *Y* will be biased since it also reflects the effect of *U*. However, one may first carry out a least squares fit of *I* on *X* and use the predicted values $\hat{X}(I)$ as predictor for *Y*. Then since $\hat{X}(I)$ is independent of *U* an unbiased effect of *X* on *Y* is obtained if the model (in particular with least squares estimation) is collapsible.

Tchetgen Tchetgen et al. (2015) and Li et al. (2015)developed instrumental variable analysis for survival data with the additive hazards model based on the collapsibility of this model. As we have seen collapsibility for Aalen regression and additive hazards regression more generally requires that covariates are independent in all risk sets. But it has also been pointed out that Cox regression will be collapsible under the same conditions. In this section it will first be demonstrated by simulation that instrumental variable analysis can be valid both for Lin-Ying and Aalen regression and for Cox regression when the data are generated by additive hazards and censoring is independent of covariates. Secondly it will be demonstrated that the instrumental variable method can be biased both for Lin-Ying and Aalen regression and for Cox regression when data are generated by a proportional hazards regression model.

#### Instrumental variables analysis under additive hazards model

The unknown confounder *U* and the instrument *I* were simulated from uniform [0, 1] distributions and the exposure was given as $X=U+I+\epsilon$ where $\epsilon$ was also drawn from a uniform [0, 1]. The $n=5000$ event times were then drawn from an exponential with hazard rates $1+X+U$ and the censoring times from a uniform [0, 1]. The instrument $\hat{X}(I)$ was generated as the fitted values from a least squares regression of *X* on *I* and so $\hat{X}(I)$ is independent of *U*. This simulation was repeated 1000 times. The proportion exact observed event times was 65%.

For each simulation three models were fitted both with Lin-Ying- and Cox regressions, first a model which is in practice impossible including both the exposure *X* and the unknown confounder *U*, then a model using only the exposure *X* and finally a model including only the instrumental variable with covariate $\hat{X}(I)$. Results from Table 5 show that both the unrealistic and the instrumental variable approach fits a value in accordance with the true $\beta _1=1$ for the Lin-Ying regression whereas the analysis with only *X* is biased. With Cox regression one similarly finds that the unrealistic analysis with *X* and *U* and the analysis with $\hat{X}(I)$ gives approximately the same results corresponding to the collapsibility property of Cox regression for independent $\hat{X}(I)$ and *U*, whereas the analysis with only *X* differs from these.

**Table 5:** Instrumental variable analysis based on event data from an additive hazards model analyzed with Lin-Ying and Cox regression

Additive hazards	Lin-Ying-regression	Cox regression
Average $\hat{\beta }_1$ in model $X+U$	1.003	0.374
Average $\hat{\beta }_1$ in model *X*	1.223	0.457
Average $\hat{\beta }_1$ in model $\hat{X}(I)$	1.006	0.376

Aalen regression models based on $\hat{X}(I)$ and *U* simultaneously, *X* alone and $\hat{X}(I)$ alone were also fitted. The upper panel of Figure S5 in the supplement gives the estimated $\hat{B}_1(t)$ for one run and the middle panel the average over 1000 $\hat{B}_1(t)$ estimates. As theory predicts there is no systematic difference between the two estimates with both *X* and *U* and with $\hat{X}(I)$, but the curve with only *X* overestimates the relation. Finally, the correlations between $\hat{X}(I)$ and *U* among those at risk at different times are plotted in the lower panel of the figure and confirms that these two terms stay uncorrelated.

#### Instrumental variables analysis under proportional hazards model

For simulation with proportional hazards for the event time the instrument *I*, the unknown confounder *U* and the exposure variable *X* were generated with same model as in the previous section. However, now the event times were drawn according to hazards $\lambda (t,x,u)=\exp (-0.8+x+u)$, i.e. with a regression coefficient for *X* equal to $\beta _1=1$. The censoring times were again drawn from a uniform [0, 1]. In each simulation the sample size was $n=5000$ and the simulations were repeated for 1000 runs. This gave a proportion exact observed events of 63%.

Table 6 presents results from the simulations for the fitted Lin-Ying and Cox regression models. It is seen that for the correct, but in practice impossible Cox regression with *X* and *U*, the true value $\beta _1=1$ is replicated. Only using *X* gives a biased result as does the analysis based on the instrument $\hat{X}(I)$. For the Lin-Ying analyses the model with *X* alone give a higher result than with *X* and *U* whereas the analysis with only the instrument $\hat{X}(I)$ give a lower result than with both *X* and *U*. These results are thus in accordance with non-collapsibility of proportional hazards models. We also note that the relative degree of bias compared to the model with both *X* ands *U* is the same for the Cox- and the Lin-Ying estimates.

**Table 6:** Instrumental variable analysis based on event data from proportional hazards model analyzed with Lin-Ying and Cox regression

Proportional hazards	Lin-Ying-regression	Cox regression
Average $\hat{\beta }_1$ in model $X+U$	2.556	1.002
Average $\hat{\beta }_1$ in model *X*	3.023	1.182
Average $\hat{\beta }_1$ in model $\hat{X}(I)$	2.270	0.885

Finally Aalen regression models were fitted both in models with *X* and *U*, only *X* and only $\hat{X}(I)$. The results are given in Figure S6 in the supplement with $\hat{B}_1(t)$ for one run in the upper panel and averaged over the 1000 runs in the middle panel. One observes that there is a difference in the curves and cumulative regression function for the unknown confounder *U* lies between those for only *X* and only $\hat{X}(I)$. The lower panel then gives correlations between *U* and $\hat{X}(I)$ among those still at risk over different times and one observes that a negative correlation develops as time increases.

## Discussion

It has in this paper been demonstrated theoretically and illustrated by means of simulation that Aalen/Lin-Ying-regression estimators and Cox regression estimators are collapsible when covariates are independent in all risks sets. The results thus gives conditions for when common exposure effects can be estimated without bias in randomized clinical studies both for additive hazards regression and for Cox-regressions. The results also gives a condition for when the simple instrumental variable analysis gives valid results again both for additive hazards and Cox-regressions.

At the same time the results shows that one should be somewhat cautious about concluding about causation from analyses of additive hazards regression, as with instrumental variable analysis, since it is generally not know that the data were generated by such a model. Furthermore, even with data generated from an additive hazards model, a correlation between covariates in risk sets may result from covariate dependent censoring patterns. This in turn can amount to biased estimation of (common) regression parameters for exposure or treatment effects when confounders are unknown and can not be accounted for.

The paper also demonstrates that it is possible to have practically uncorrelated covariates in all risk sets even when the data are generated by a proportional hazards model if censoring counters the model induced dependence and suggested that truncation patterns can generate a similar structure. Other mechanisms that may generate uncorrelated covariates in risk sets can be time-dependency of covariates $z_i(t)$ and time-dependency of effects, e.g. an extended proportional hazards model with regression functions $\beta _j(t)$. It may be considered that such structures are not very common in practice. Nevertheless, the condition of independence of covariates in all risk sets could provide a background for evaluating the potential for estimation of unbiased regression parameters.

It should be mentioned that the present work was not set out to be a discussion of estimation of treatment or causal effects. Rather, the start point was related to considerations of matched cohort studies. In such studies one will study a subset of the entire cohort obtained by, for each individual with a particular (often rare) exposure, selecting a small number unexposed reference individuals. These are matched, i.e. chosen equal to the index (exposed) individual, on a number of known and otherwise potentially confounding variables. Thus the matched data are made balanced and the exposure and confounders are initially uncorrelated. The idea was to investigate the use of additive hazards regression where confounders are ignored in the analyses. However, it turned out that this did not work well if the data were in fact generated by proportional hazards models.

## Supplementary Information

Below is the link to the electronic supplementary material.

Supplementary file 1 (PDF 277 kb)

## References

[CR1] Aalen OO, Klonecki W, Kozek A, Rosinski J (1980). A model for nonparametric regression analysis of counting processes. Mathematical statistics and probability theory Vol. 2. Lecture notes in statistics.

[CR2] Aalen OO (1989). A linear-regression model for the analysis of life times. Stat Med.

[CR3] Aalen OO, Borgan Ø, Gjessing HK (2008). Survival and event history analysis: a process point of view.

[CR4] Aalen OO, Cook RJ, Røysland K (2015). Does Cox analysis of a randomized survival study yield a causal treatment effect?. Lifetime Data Anal.

[CR5] Andersen PK, Gill RD (1982). Cox’s regression model for counting processes: a large sample study. Ann Stat.

[CR6] Bretagnolle J, Huber-Carol C (1988). Effects of omitting covariates in Cox’s model for survival data. Scand J Stat.

[CR7] Cox DR (1972). Regression models and life-tables (with discussion). J R Stat Soc Ser B.

[CR8] Gail MH, Wieand S, Piantadosi S (1984). Biased estimates of treatment effect in randomized experiments with nonlinear regression and omitted covariates. Biometrika.

[CR9] Greenland S, Robins JM, Pearl J (1999). Confounding and collapsibility in causal inference. Stat Sci.

[CR10] Henderson R, Milner A (1991). Aalen plots under proportional hazards. J R Stat Soc Ser C Appl Stat.

[CR11] Li J, Fine J, Brookhart A (2015). Instrumental variable additive hazards models. Biometrics.

[CR12] Lin DY, Ying Zl (1994). Semiparametric analysis of the additive risk model. Biometrika.

[CR13] McKeague IW, Sasieni PD (1994). A partly parametric additive risk model. Biometrika.

[CR14] Martinussen T, Scheike TH (2006). Dynamic regression models for survival data.

[CR15] Struthers CA, Kalbfleisch JD (1986). Misspecified proportional hazard models. Biometrika.

[CR16] Solomon PJ (1984). Effect of misspecification of regression models in the analysis of survival data. Biometrika.

[CR17] Tchetgen Tchetgen EJ, Walter S, Vansteelandt S, Martinussen T, Glymour M (2015). Instrumental variable estimation in a survival context. Epidemiology.

[CR18] Whittemore AS (1978). Collapsibility of multidimensional contingency tables. J R Stat Soc Ser B.

