# Supplementary material for: Cox regression can be collapsible and Aalen regression can be non-collapsible
Source: Lifetime Data Anal. 2022 Oct 21;29(2):403–19. doi: 10.1007/s10985-022-09578-0 (PMC10006274; doi:10.1007/s10985-022-09578-0)
Supplement: Supplementary file 1 — (PDF 277 kb) [file 10985_2022_9578_MOESM1_ESM.pdf]

Supplement to "Cox-regression can be collapsible and Aalen-regression can be non-collapsible", Lifetime Data Analysis D-22-00017-Revision 1, Special issue for Ørnulf Borgan.

This supplement contains plots of cumulative regression functions estimated by Aalen regression from the simulation studies in Section 3 of the paper. The 6 different simulation models were generated with population sizes $n = 5000$ and repeated for 1000 runs.

For Figure 1 data were generated by a proportional hazards model with censoring independent of covariates as described in Section 3.1.1.

For Figure 2 the data were generated by an additive hazards model with censoring independent of covariates as described in Section 3.1.2.

For Figure 3 the data were generated by a proportional hazards model with censoring dependent of covariates as described in Section 3.2.1.

For Figure 4 the data were generated by an additive hazards model with censoring dependent of covariates as described in Section 3.2.2.

For Figure 5 the data were generated by an instrumental variables setup with event times from a proportional hazards model as described in Section 3.3.1.

For Figure 6 the data were generated by an instrumental variables setup with event times from an additive hazards model as described in Section 3.3.2.

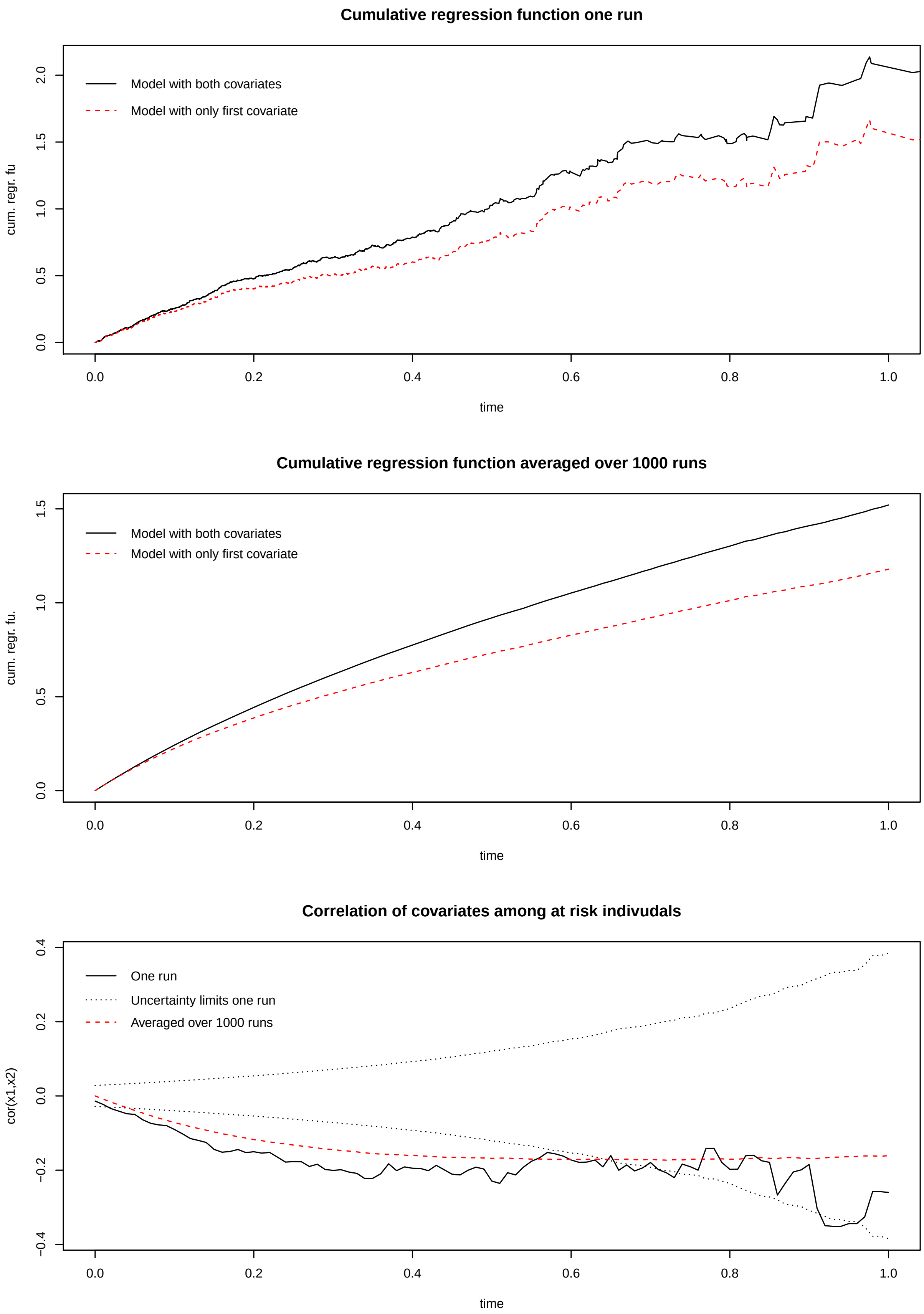

Fig. 1 Cumulative incidence functions $B_1(t) = \int_0^t \beta_1(s)ds$ in models with both covariates and only z_1 in one run (Panel A) and averaged over 1000 runs (Panel B). Panel C gives the correlation between z_1 and z_2 over individuals still at risk. The data were generated by a proportional hazards model with censoring independent of covariates

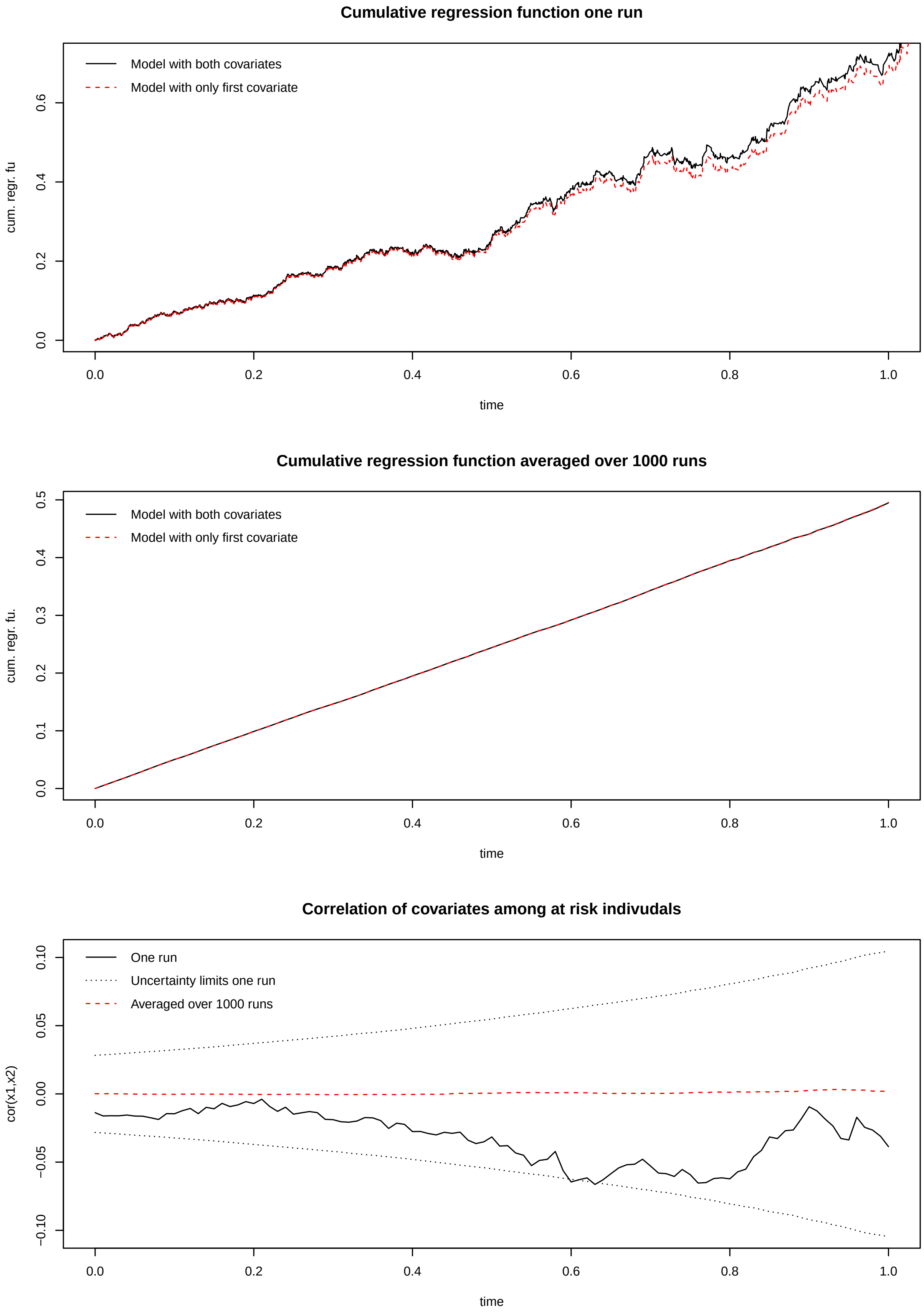

Fig. 2 Cumulative incidence functions $B_1(t) = \int_0^t \beta_1(s)ds$ in models with both covariates and only z_1 in one run (Panel A) and averaged over 1000 runs (Panel B). Panel C gives the correlation between z_1 and z_2 over individuals still at risk. The data were generated by Lin-Ying models with censoring independent of covariates

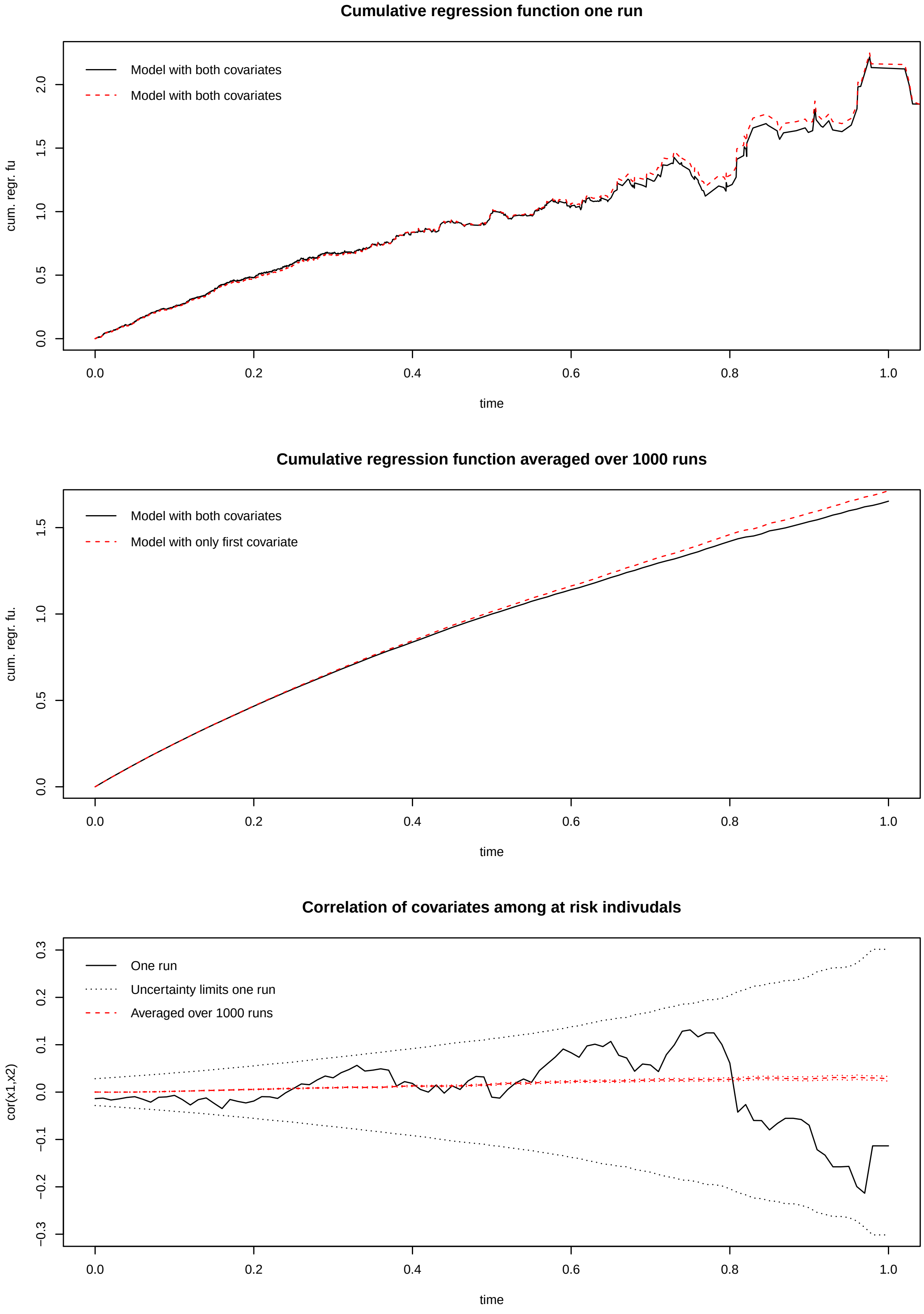

Fig. 3 Cumulative incidence functions $B_1(t) = \int_0^t \beta_1(s)ds$ in models with both covariates and only z_1 in one run (Panel A) and averaged over 1000 runs (Panel B). Panel C gives the correlation between z_1 and z_2 over individuals still at risk. The event and censoring times were generated with proportional hazards models

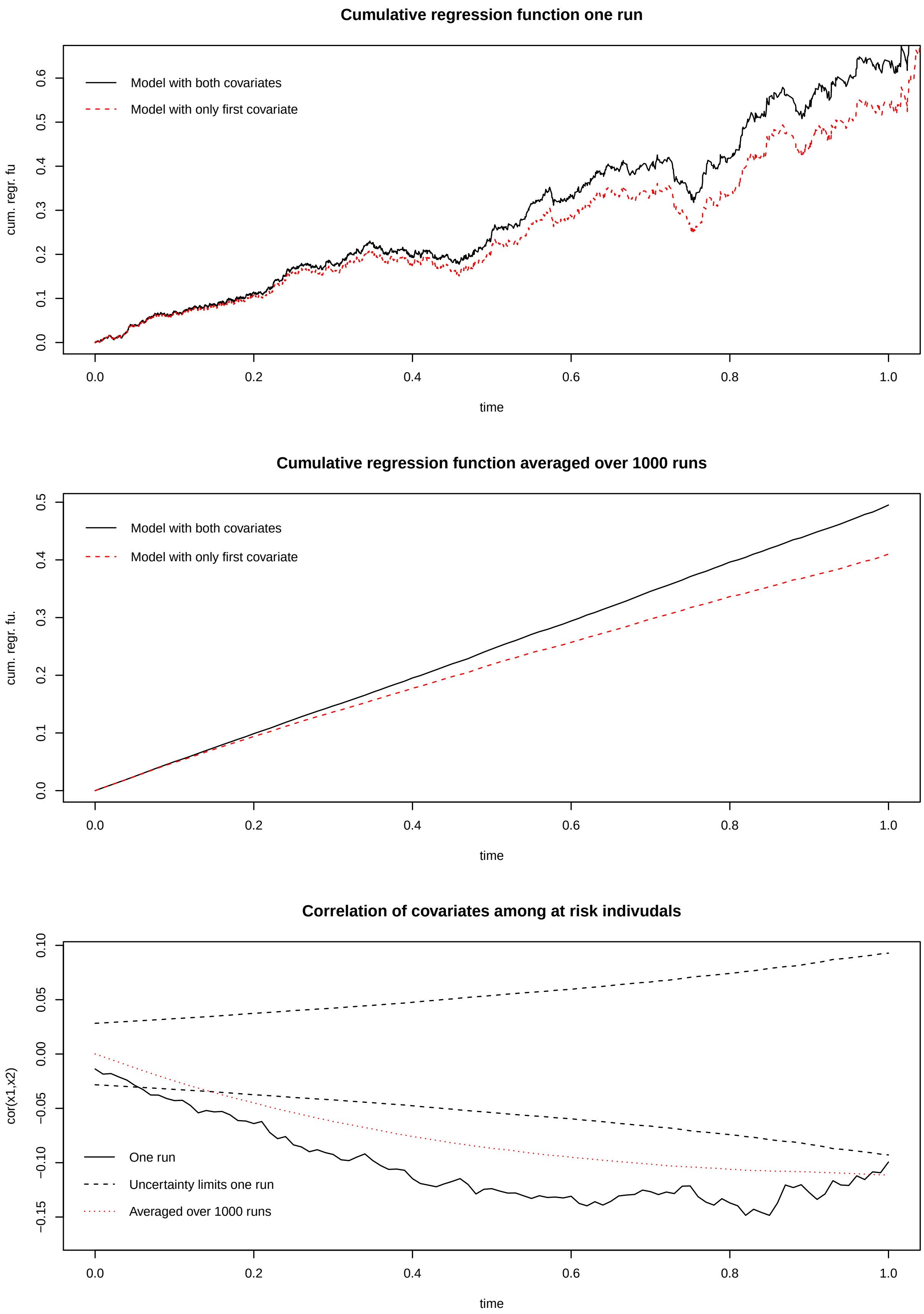

Fig. 4 Cumulative incidence functions $B_1(t) = \int_0^t \beta_1(s)ds$ in models with both covariates and only z_1 in one run (Panel A) and averaged over 1000 runs (Panel B). Panel C gives the correlation between z_1 and z_2 over individuals still at risk. The event times were generated by an additive hazards model while the censoring times were drawn according to a proportional hazards model

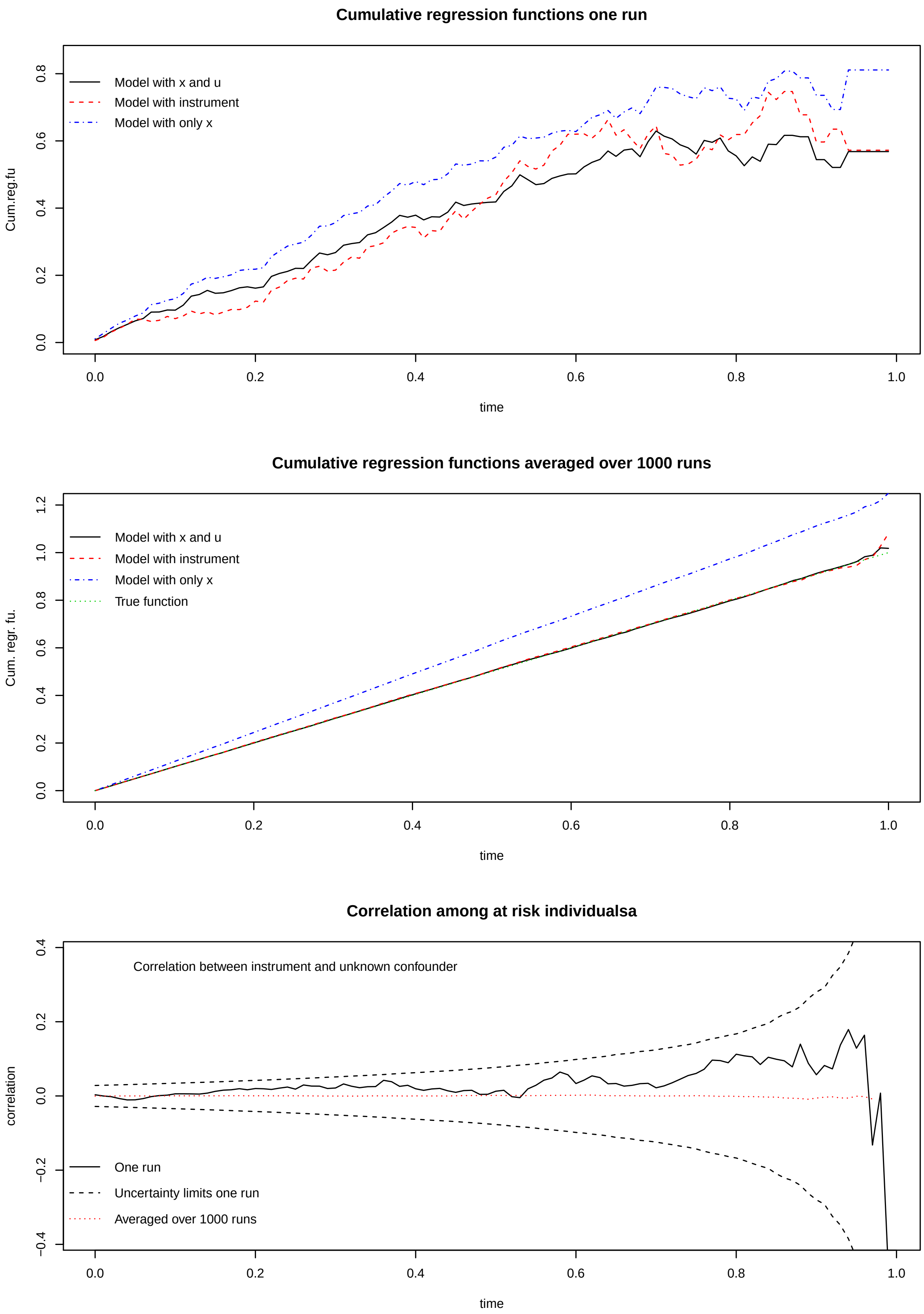

Fig. 5 Cumulative incidence functions $B_1(t) = \int_0^t \beta_1(s)ds$ in models with both X and U and only $\hat{X}(I)$ in one run (Panel A) and averaged over 1000 runs (Panel B) under additive hazards models for X and U. Panel C gives the correlations between the unknown confounder U and the instrument $\hat{X}(I)$ over those still at risk. The event times are drawn from a Lin-Ying model and censoring is independent of covariates

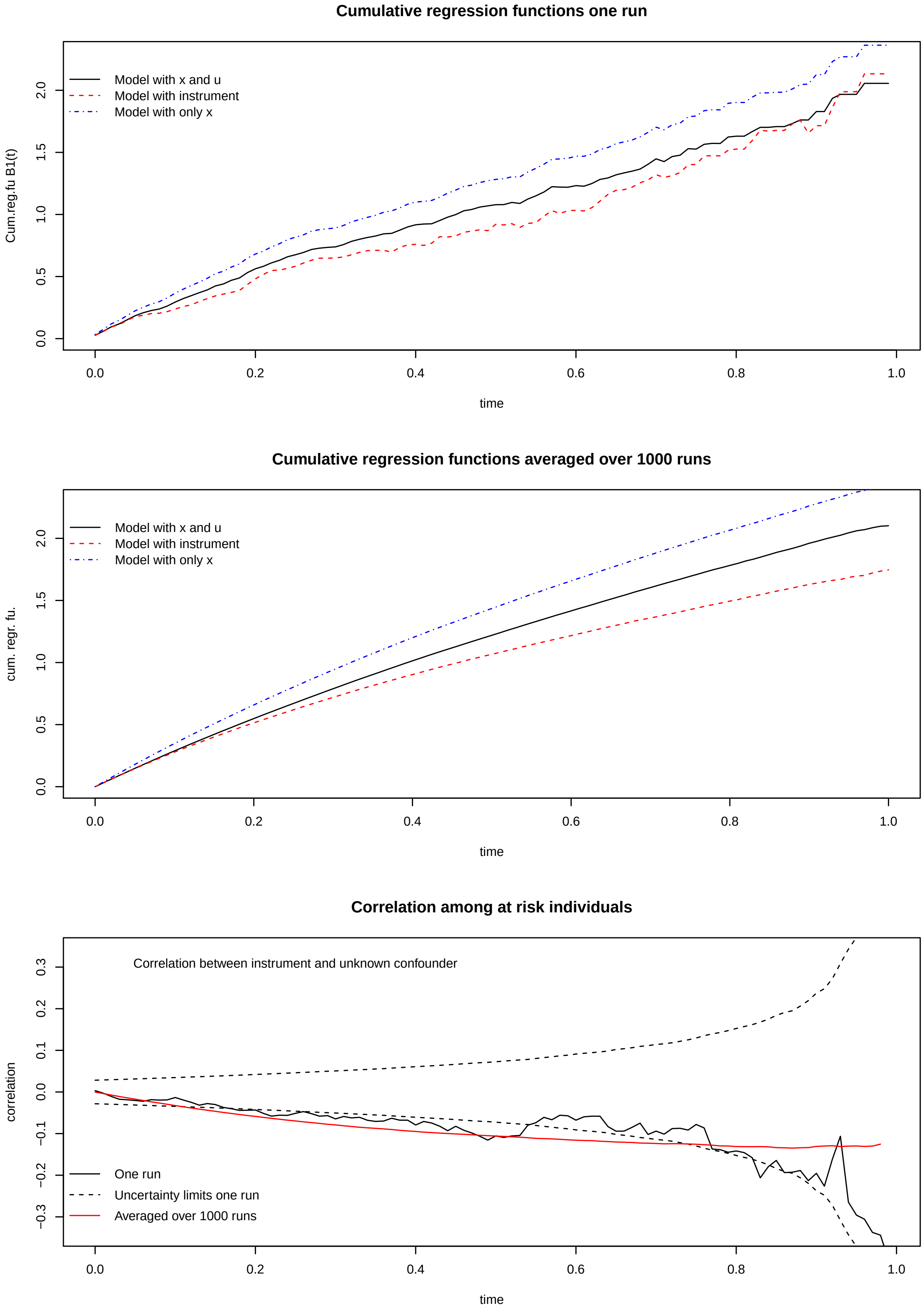

Fig. 6 Cumulative incidence functions $B_1(t) = \int_0^t \beta_1(s)ds$ in models with both X and U and only $\hat{X}(I)$ in one run (Panel A) and averaged over 1000 runs (Panel B) under proportional hazards models for X and U. Panel C gives the correlation between the unknown confounder U and the instrument $\hat{X}(I)$ over those still at risk. The event times were drawn from a proportional hazards model and censoring was independent of covariates
